# Shoulder Arthroplasty as a Day Case: Is It Better? †

**DOI:** 10.3390/jcm12123886

**Published:** 2023-06-07

**Authors:** Syed Mohammed Taif Rizvi, Benjamin Lenane, Patrick Lam, George A. C. Murrell

**Affiliations:** Orthopaedic Research Institute, The St George Hospital, Level 2, 4-10 South Street, Kogarah, NSW 2217, Australia

**Keywords:** shoulder arthroplasty, ambulatory care, day surgery, surgical complications, post-operative pain, revision surgery

## Abstract

**Introduction**: A retrospective case-controlled study was performed to evaluate the outcomes of shoulder arthroplasty performed as a day case in carefully selected patients, compared to the traditional inpatient approach. **Materials and Methods**: Patients who had total or hemiarthroplasty of the shoulder performed as a day case or inpatient procedure were recruited. The primary outcome compared rates of uneventful recovery, defined by the absence of complications or readmission to the hospital within six months of surgery, between the inpatient and outpatient groups. Secondary outcomes included examiner-determined functional and patient-determined pain scores at one, six, twelve, and twenty-four weeks post-surgery. A further assessment of patient-determined pain scores was carried out at least two years post-surgery (5.8 ± 3.2). **Results**: 73 patients (36 inpatients and 37 outpatients) were included in the study. Within this time frame, 25/36 inpatients (69%) had uneventful recoveries compared to 24/37 outpatients (65%) (*p* = 0.17). Outpatients showed significant improvement over pre-operative baseline levels in more secondary outcomes (strength and passive range-of-motion) by six months post-operation. Outpatients also performed significantly better than inpatients in external rotation (*p* < 0.05) and internal rotation (*p* = 0.05) at six weeks post-surgery. Both groups showed significant improvement compared to pre-operative baselines in all patient-determined secondary outcomes except the activity level at work and sports. Inpatients, however, experienced less severe pain at rest at six weeks (*p* = 0.03), significantly less frequent pain at night (*p* = 0.03), and extreme pain (*p* = 0.04) at 24 weeks, and less severe pain at night at 24 weeks (*p* < 0.01). By a minimum of two years post-operation, inpatients were more comfortable repeating their treatment setting for future arthroplasty (16/18) compared to outpatients (7/22) (*p* = 0.0002). **Conclusions**: At a minimum of two years of follow-up, there were no significant differences in rates of complications, hospitalizations, or revision surgeries between patients that underwent shoulder arthroplasty as an inpatient versus an outpatient. Outpatients demonstrated superior functional outcomes but reported more pain at six months post-surgery. Patients in both groups preferred inpatient treatment for any future shoulder arthroplasty. **What is Known About This Subject:** Shoulder arthroplasty is a complex procedure and has traditionally been performed on an inpatient basis, with patients admitted for six to seven days post-surgery. One of the primary reasons for this is the high level of post-operative pain, usually treated with hospital-based opioid therapy. Two studies demonstrated outpatient TSA to have a similar rate of complications as inpatient TSA; however, these studies only examined patients within a shorter-term 90-day post-operative period and did not evaluate functional outcomes between the two groups or in the longer term. **What This Study Adds to Existing Knowledge:** This study provides evidence supporting the longer-term results of shoulder arthroplasty done as a day case in carefully selected patients, which are comparable to outcomes in patients that are admitted to the hospital post-surgery.

## 1. Introduction

Elective shoulder arthroplasty is an increasingly common surgical intervention for the treatment of degenerative pathologies of the shoulder joint since the first shoulder arthroplasty was performed by Péan in the 1890s [1]. Shoulder arthroplasty is a complex procedure and has traditionally been performed on an inpatient basis, with patients admitted six to seven days post-surgery. One of the primary reasons for this is the high level of post-operative pain, usually treated with hospital-based opioid therapy [2,3]. Evidence suggests that early rehabilitation can improve functional outcomes after shoulder arthroplasty, adding to the importance of post-operative pain control [4].

Recent developments in surgical and anesthetic techniques, however, have raised the possibility of performing ambulatory shoulder arthroplasty. There is a growing trend in developed nations to perform more surgeries as outpatient procedures, including many orthopedic procedures [5]. However, to date, this expansion in orthopedics has largely been limited to arthroscopic procedures; total shoulder arthroplasty (TSA) and hemi-shoulder arthroplasty (HSA) are still largely performed in an inpatient setting. Outpatient surgery is economically advantageous for both hospitals and individual patients and may allow for a better distribution of hospital resources [6,7]. One study from the United States has shown that outpatient total shoulder arthroplasty can save between USD 747 and USD 15,507 per patient compared to inpatient management and a total annual saving between USD 4.1M and USD 349M [8]. The practicality of performing TSA and HAS in an outpatient setting was established when, in 2006, Ilfeld et al. demonstrated the efficacy of continuous interscalene nerve block (CISB) to control pain in patients discharged to their homes after shoulder arthroplasty [2] and in 2008 when, in a retrospective study of 16 patients, Gallay et al. reported the successful implementation of a regional model of care for performing shoulder arthroplasty as a day case with adequate post-operative care and analgesia [4]. Two studies demonstrated outpatient TSA to have a similar rate of complications as inpatient TSA; however, these studies only examined patients within a shorter-term 90-day post-operative period and did not evaluate functional outcomes between the two groups or in the long term [5,9,10]. The aim of this study, therefore, was to assess the complication rates of performing shoulder arthroplasty on an outpatient basis through a larger, longer-term comparison of patient outcomes after day surgery vs. a traditional inpatient approach and to compare shoulder function and pain in these patients. Our hypothesis was that there would be no significant difference between inpatient and outpatient arthroplasty with respect to complication rates, post-operative pain, and functional outcomes.

## 2. Materials and Methods

A retrospective cohort study assessed the feasibility of performing shoulder arthroplasty as an outpatient procedure. The main outcome assessed was the complication rate, widely defined as any deviation from a standard, uneventful post-operative recovery and classified as major or minor depending on their effect on long-term outcome. Secondary outcomes included physician-determined functional scores concerning shoulder strength and range of motion and patient-assessed pain scores per the L’Insalata Shoulder Questionnaire [11]. All patients undergoing primary total or hemiarthroplasty of the shoulder performed by a single surgeon (G.A.C.M.) between January 2004 and July 2012 were included in the study. Approval was obtained from the appropriate institutional ethics review board, and informed consent for data collection was obtained at the first visit. Patients were assessed preoperatively and were followed up post-operatively face-to-face at one week, six weeks, twelve weeks, and six months. At two years post-surgery, a further follow-up was conducted by telephone.

Inclusion criteria included primary shoulder arthroplasty performed by a single surgeon between 2004 and 2012 for osteoarthritis and other arthropathies. Revision surgeries were excluded, and reverse total shoulder arthroplasty procedures were also excluded, as these have different indications and complication rates compared to primary shoulder arthroplasty being performed to treat arthritis [12]. Patients with less than the minimum six months follow-up were also excluded. All subjects meeting these criteria were divided into two cohorts at the time of the preadmission clinic, an inpatient group of patients admitted to the hospital for a minimum of one night following surgery and an outpatient group of patients discharged on the day of their surgery. No randomization was used in this study. Initially, only a small number of patients were treated on an outpatient basis. Following the completion in early 2007 of a new facility specializing in day surgery, a decision was made to perform more surgeries as day cases. Thereafter, the approach was to perform surgeries as day cases unless this was contraindicated by patient factors such as illness, multiple comorbidities, patient request, or failure to comply with pre-operative instructions. Intention-to-treat analysis was used.

Pre-operative care

Preoperatively, patients underwent an examination by the principal surgeon. This included clinical history, physical examination, investigations, and the use of two standardized questionnaires, one patient-reported and one examiner-determined. The patient-reported questionnaire is based on the L’Insalata Self-Administered Shoulder Questionnaire, with proven validity and reliability as a clinical tool for assessing pain and shoulder function [8]. In this questionnaire, patients provide a rating of 1 to 5 for six questions related to pain, stiffness, level of function, and overall satisfaction. In addition, two questions on the level of activity at work and sport are rated from 1 to 4, with 4 being the highest level. The physical examination involved the assessment of a passive range of motion (ROM) by visual estimation and strength testing with the use of a handheld dynamometer, both assessment techniques which have been previously validated [13,14,15].

Peri-operative care

All patients were reviewed by the principal surgeon and anesthetist prior to surgery. For all patients, the procedure was carried out under interscalene local anesthesia, in the form of injected ropivacaine or equivalent agent, in conjunction with general sedation. Antibiotic prophylaxis was administered intravenously on induction of anesthesia. Surgery was performed in the beach chair position with a standard deltopectoral approach. Tournier–Aqualis prostheses were used in both total and hemiarthroplasties.

Post-operative care

Post-operatively, patients were observed in the post-anesthesia care unit and prescribed oral analgesia in the form of a paracetamol (1000 mg) and codeine phosphate (60 mg) combination and/or tramadol (50–100 mg). Patients in the inpatient group were discharged to the ward. There, they began early rehabilitation range-of-motion exercises. During this period, any complications that arose were documented. Patients were discharged to their homes after a minimum admission of one night. Patients in both groups were placed in a shoulder immobilizer sling and a Cryo/Cuff cooling device to be worn for 48 h after surgery. All patients were encouraged to begin passive range-of-motion exercises from post-operative day one.

Patients in the outpatient group were discharged to their homes from the recovery bay. Patients were instructed not to drive themselves home and to stay home with a family member/carer for at least the first 24 h. These patients were given standardized written information for aftercare and instructions for returning to the hospital if required, including a contact phone number.

At one week, six weeks, twelve weeks, and six months patients returned for follow-ups where both patient-determined and examiner-determined assessment was utilized as with the pre-operative visit. At least two years after surgery, patients were contacted by an examiner who conducted a phone survey utilizing questions from the patient-determined questionnaire, in addition to several further questions on complications after six months, readmissions to the hospital, and any GP visits after six months (Supplementary Materials).

### Statistical Analysis

Data were analyzed in an intention-to-treat fashion, meaning all patients were assessed in the groups to which they were assigned. The inpatient group was compared to the outpatient group at each time point.

For non-parametric data such as pain scores and internal rotation range of motion (vertebral levels), the Mann–Whitney Rank Sum test was used to assess differences between inpatients and outpatients at each time point.

For parametric data such as shoulder strength and range of motion, the unpaired Student’s t-test was used to assess differences between the two groups at each time point, with a significance level set at 0.05. The paired Student’s t-test assessed differences within each group between pre-operative and six-month post-operative time points.

A two-way ANOVA with Bonferroni corrections was used to assess two factors (the effect of time and mode of discharge) between pre-operative and follow-up time points.

Chi-square analysis assessed dichotomous data, such as patient demographics and the presence or absence of complications. Statistical analysis was performed using SigmaPlot v11 (Systat Software, Inc. Chicago, IL, USA) with a significance level set at 0.05.

## 3. Results

Complete data sets were available for 73 patients who had a shoulder arthroplasty between 2004 and 2012, with a minimum six-month follow-up, and of these, 40 patients had a minimum of two years follow-up (average long-term follow-up 5.8 years ± 3.2, range 2–9). There were 36 patients in the inpatient group and 37 in the outpatient group. Both groups were well-matched in age, gender, type, and duration of surgery (Table 1). A total of 72% (26/36) of inpatients and 68% (25/37) of outpatients underwent total shoulder arthroplasty. Additionally, 28% of inpatients (10/36) and 32% (12/37) of outpatients underwent hemiarthroplasty. All procedures were indicated for osteoarthritis. Operation time for inpatients was 93 ± 23 min (mean ± standard deviation), and for day cases, 92 ± 29 min. Outpatients had a similar duration of symptoms prior to surgery. Of this starting cohort, 22 patients in the inpatient group and 18 in the outpatient group responded to follow-up attempts at least two years after surgery. There were no significant differences between the inpatient and outpatient groups at long-term follow-up.

### 3.1. Primary Outcome

**Figure 1:**
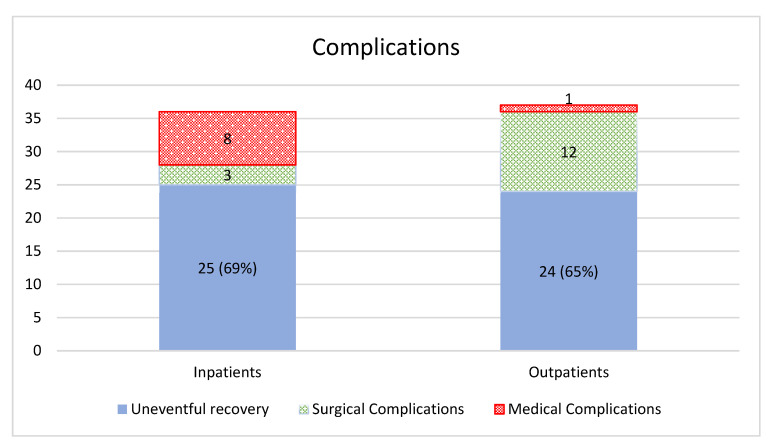
Number of complications in inpatient group (*n* = 36) vs. outpatient group (*n* = 37) at six months follow-up (*p* = 0.17).

The primary outcome for the study was uneventful recovery, defined as nil hospitalizations or complications reported within six months (the duration of standard post-operative follow-up at our institution). Within this time frame, 25/36 inpatients (69%) had uneventful recoveries compared to 24/37 outpatients (65%) (*p* = 0.17) (Figure 1).

Of the 11 inpatients who experienced complications within six months (Table 2), there were three orthopedic complications (3/11, 27%). One patient had an intraoperative bleed, and hemiarthroplasty was performed instead of the planned total shoulder arthroplasty. One patient from the inpatient group suffered from a ruptured long head of biceps within a year of the operation. One patient dislocated their shoulder six weeks after surgery. There were no reoperations.

The remainder of the complications in the inpatient group were medical complications (8/11, 73%). Two patients had an extended admission due to fever and two more for slight nausea in recovery. Other complications in the inpatient group included post-operative admission for dyspnoea, disorientation, supplementary oxygen requirement, and one precautionary case of dysrhythmia detected during the operation, with investigations being normal.

Of the 13-day case (ambulatory care) patients who experienced complications (Table 3), the majority were surgical complications (12/13, 92%).

There were two cases of infection, one superficial skin infection treated with surface debridement and one *staphylococcus aureus* infection, which required debridement, a washout of the joint, and intravenous flucloxacillin treatment. One patient had a hematoma drained within two months of surgery. One patient suffered weakness and paraesthesia over the ulnar nerve distribution, presumed to have been caused by damage to the brachial plexus during placement of the interscalene block. Another suffered a radial nerve palsy and Dupuytren’s contracture, which resolved within six months. In one case, hemiarthroplasty was performed instead of total arthroplasty after a surgical bleed. One patient fell onto the shoulder two months after surgery, received arthroscopic debridement and gentle manipulation under anesthetic a month later, and eventually underwent revision total shoulder arthroplasty two years after the original operation. Another patient suffered an episode of wrist extension and elbow flexion dysfunction with associated C6/C7 numbness. Other complications in the outpatient group included readmission one day after release for hypotension, one case of bruising and persistent post-operative pain and bruising, one case of trapezius discomfort, and one case of post-operative stiffness. The sole medical complication (1/13, 8%) was one recurrent syncopal episode within a month of surgery. There were four reoperations in total.

Of patients contacted at two years post-surgery, 5% (1/22) of inpatients and 11% (2/18) of outpatients reported readmission to the hospital within six months of their operation (*p* = 0.43), and there was no statistically significant difference. One outpatient reported admission to the hospital for problems with the treated shoulder after six months post-surgery, whereas zero inpatients required readmission. One outpatient in the long-term follow-up cohort underwent revision surgery, whereas nil inpatients had required revision surgery. At long-term follow-up, 39% (7/18) of outpatients reported GP visits concerning their treated shoulders, compared with 9% (2/22) of inpatients (*p* = 0.02).

### 3.2. Secondary Outcomes

#### 3.2.1. Passive Range of Motion

From pre-operative assessment to six-month follow-up, patients who underwent shoulder arthroplasty as inpatients improved significantly in abduction (*p* = 0.005), external rotation (*p* = 0.0002), and internal rotation (*p* = 0.045) passive range of motion but showed no statistically significant improvement in forward flexion. Outpatients showed statistically significant improvement in all movements of the passive range of motion: abduction (*p* = 0.01), external rotation (*p* < 0.0001), internal rotation (*p* = 0.001), and forward flexion (*p* = 0.02).

Outpatients achieved 41 ± 4° of external rotation at six weeks after surgery, compared to 31 ± 3° of external rotation achieved by inpatients at the same time point (*p* < 0.05). A two-way ANOVA analysis, however, suggested that time was the only significant factor in this difference (*p* = 0.007).

**Figure 2:**
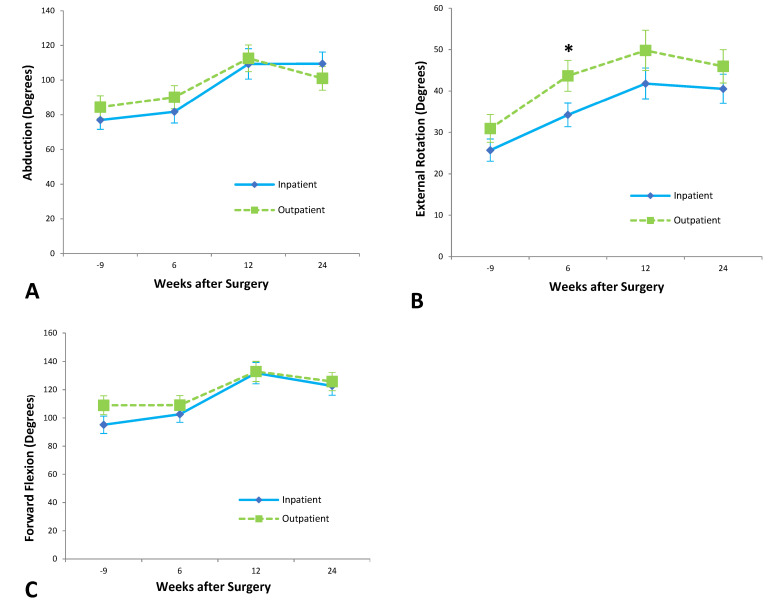
Shoulder range of motion in (**A**) abduction, (**B**) external rotation, and (**C**) forward flexion. * *p* < 0.05.

At six weeks, outpatients had achieved a score of 5 ± 1 on internal rotation (corresponding with vertebral level L5), significantly better than the inpatient score of 3 ± 1 (S2) (*p* = 0.05) (Figure 2).

#### 3.2.2. Strength

Patients in the inpatient group demonstrated a significant improvement in lift-off strength (*p* = 0.03) but did not show any statistically significant improvement over baseline measurements in supraspinatus strength, adduction strength, internal rotation, or external rotation strength within six months of surgery. At six months post-surgery, the outpatient group had improved significantly in internal rotation (*p* = 0.01), external rotation (*p* < 0.001), and adduction strength (*p* = 0.03) compared to pre-operative levels. Outpatients showed no significant improvement in supraspinatus or lift-off strength.

**Figure 3:**
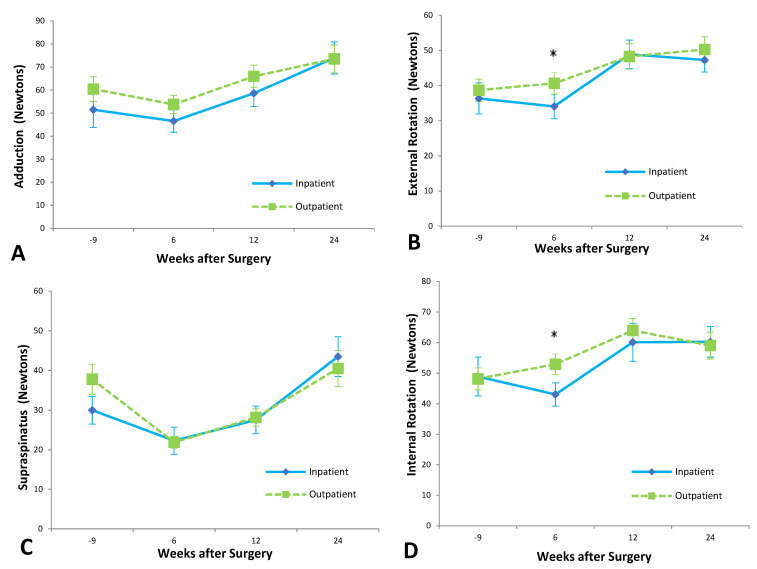
Shoulder strength in (**A**) adduction, (**B**) external rotation, (**C**) supraspinatus, and (**D**) internal Rotation. There were nil significant differences at any of the time points. * *p* < 0.05.

At six weeks’ follow-up, outpatients were significantly stronger than inpatients. External rotation strength at six weeks for the outpatient group was 39 ± 4 N, while inpatient strength was 29 ± 4 N (*p* = 0.05) (Figure 3). Outpatients were significantly stronger on internal rotation six weeks after surgery, with a mean strength of 52 ± 4 N compared to a mean inpatient strength of 39 ± 4 N (*p* = 0.03).

At twelve weeks after surgery, outpatients were significantly stronger than inpatients on lift-off testing. Lift-off strength in the outpatient group was 26 ± 4 N, significantly higher than the inpatient strength of 14 ± 4 N (*p* = 0.05). A two-way ANOVA analysis indicated patient discharge as the main cause of this difference (*p* = 0.010), with subject matching playing a less important role (*p* = 0.047) and being time insignificant.

#### 3.2.3. Patient-Determined Outcomes

From pre-operative to post-operative, inpatients showed a significant improvement in frequency of extreme pain, pain at night, and pain with activity; severity of pain at rest, at night, and with overhead activity; difficulty with overhead activity, and activity behind the back; stiffness, and overall shoulder rating. In all of these outcomes, *p* < 0.0001 except for difficulty with overhead activity, where *p* = 0.0002.

Outpatients likewise showed a significant improvement in all the above outcomes, with *p* < 0.0001 for all outcomes except difficulty with overhead activity (*p* = 0.010) and difficulty with activity behind the back (*p* = 0.010).

**Figure 4:**
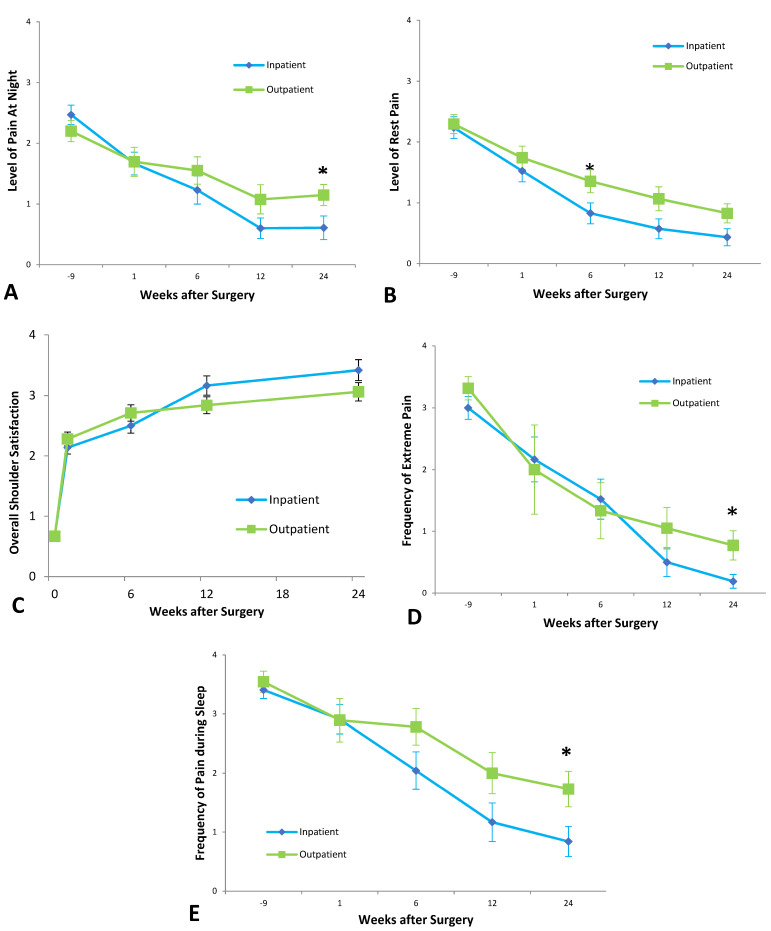
Patient ranked outcomes, including (**A**) level of pain at night, (**B**) level of rest pain, (**C**) overall shoulder satisfaction, (**D**) frequency of extreme pain, and (**E**) frequency of pain during sleep. Statistically significant differences are indicated by asterisks (* *p* < 0.05).

Six weeks after surgery, the outpatient group experienced more pain at rest than inpatients, with a mean pain score between ‘Moderate’ and ‘Mild,’ significantly higher than the inpatient mean score just below ‘Mild’ (*p* = 0.0346) (Figure 4).

At six-months follow-up, outpatients experienced more frequent and more severe pain at night. Outpatients had a mean of ‘Weekly’ night pain, significantly higher than the inpatient mean of ‘Monthly’ (*p* = 0.0278). For the outpatient group, mean pain scores for pain at night were above ‘Mild,’ significantly higher than the inpatient mean between ‘Mild’ and ‘None’ (*p* = 0.0093).

Outpatients also experienced extreme pain more frequently at six months post-surgery. The outpatient group had a mean of ‘Monthly’ extreme pain at six months after surgery, compared to a mean of ‘Never’ for the inpatient group (*p* = 0.04). A two-way ANOVA analysis suggested that patient discharge was not a significant source of variation (*p* = 0.05), with only time significance (*p* < 0.0001). When asked to rank overall shoulder satisfaction, there was no significant difference between the two groups at any time point.

Outpatients had a higher frequency of pain at rest at six months compared to inpatients. There was no significant difference with regard to the frequency of extreme pain at any timepoints.

#### 3.2.4. Discharge Preference at Minimum Two-Years Follow-Up

**Figure 5:**
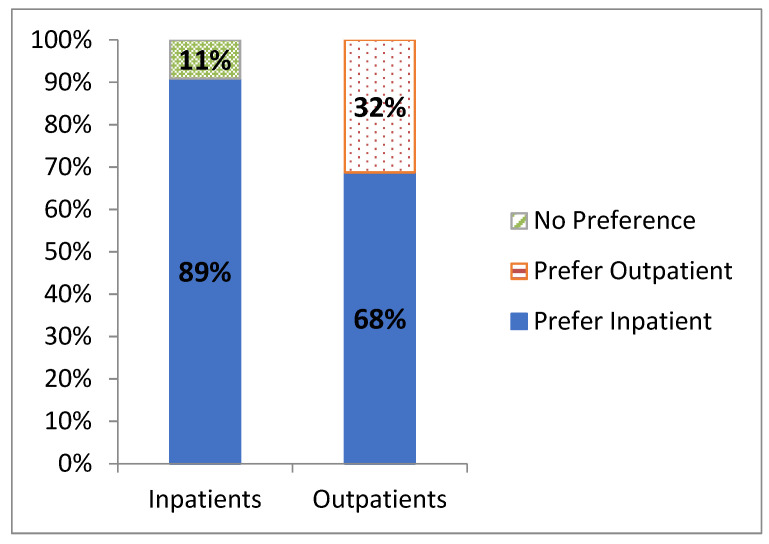
Patient preferred discharge expressed as percentages in inpatient (*n* = 18) and outpatient (*n* = 22) groups at one-year follow-up.

At a minimum of two-year follow-up, the majority of the inpatient group (16/18, 89%) indicated that they would prefer inpatient admission after any future shoulder arthroplasty (Figure 5), with the remaining 11% (2/18) having no clear preference. In the outpatient group, 32% (7/22) indicated a preference for outpatient surgery, but the remaining 68% (15/22) expressed a preference for inpatient admission. Compared to outpatients, inpatients were more comfortable repeating their treatment setting for future arthroplasty (16/18) compared to outpatients (7/22) (*p* = 0.0002).

## 4. Discussion

Performing shoulder arthroplasty as a day case in selected patients resulted in a complication rate similar to that of the traditional inpatient approach by six months post-operatively. There were no significant differences between the inpatient and outpatient groups with respect to the need for revision surgery or readmission to the hospital within a minimum of two years post-surgery. Compared to inpatients, outpatients visited a GP with regards to their shoulder more frequently, had an inferior pain experience, and expressed a preference for inpatient surgery if for future arthroplasty. Despite these differences, our data showed that having shoulder arthroplasty as an inpatient or an outpatient was not associated with significant differences in long-term complication rates.

Regarding complication rates between the two groups, our findings are consistent with the other literature. Cimino et al. [16] performed a meta-analysis that demonstrated no significant difference between inpatients and outpatients in complications, readmission, revision, or infection. Leroux et al. [17] also did not note any statistically significant differences between these groups regarding 30-day complication rates and noted high patient satisfaction within their outpatient cohort.

In our study, both groups experienced similar improvements in range of motion and strength, with the outpatient group having greater strength and range of motion at six weeks. Both inpatient and outpatient groups achieved a significant reduction in pain frequency and severity and continued to improve beyond six months. However, outpatients did experience a higher frequency of extreme pain at six months and night by six months. There was also a trend at six months post-surgery towards increased pain with overhead movements, increased pain with activity, and a widening difference in overall shoulder satisfaction in outpatients compared to inpatients at this time point. Notably, these disparities between inpatients and outpatients at the 6-month timepoint appear to have largely resolved by one-year post-surgery.

At long-term follow-up, 39% (7/18) of outpatients reported GP visits concerning their treated shoulders, compared with just 9% (2/22) of inpatients (*p* = 0.02). Furthermore, most patients expressed a preference to have future arthroplasty in an inpatient admission. It is possible that patients who experienced peri-operative complications would opt for an inpatient route in their next admission in their hindsight, as it may be perceived as the “safer option” given the longer period of post-operative monitoring and care. More notable is that of patients who underwent their procedure as an outpatient, 68% preferred future arthroplasty to occur in an inpatient setting. This suggests that while outpatient arthroplasty yields excellent functional outcomes, many patients were not fully satisfied with their care in this treatment setting. This finding raises the question of what can be done to improve the patient experience in this group. This difference in patient satisfaction may possibly be related to the outpatient group experiencing worse pain in the early postoperative period, as reflected by these patients requiring additional visits to the GP with concerns about their shoulder. Rauck et al. [18] performed a retrospective review of satisfaction in patients that underwent reverse shoulder arthroplasty and determined that satisfaction post-operation was strongly correlated with improvements in pain and outcomes scores. Menendez et al. [19] reported that the strongest predictors of severe postoperative pain post shoulder arthroplasty included pre-operative chronic opioid use and depression; they concluded that addressing psychological and social determinants of health may make a significant difference in the pain experience post-operation. Therefore, frequent outpatient evaluation and management of these patients’ mental health and pain may be useful in ensuring greater pain control and patient satisfaction in patients undergoing outpatient shoulder arthroplasty. Additionally, they also noted that patients reporting severe pain stayed longer in the hospital (2.9 days vs. 2.0 days) compared to those with pain <75th percentile. Therefore, patients who are at risk of severe postoperative pain may not be ideal candidates for day arthroplasty and may require a longer admission than other patients.

### Limitations

An important limitation of our study is that as a retrospective cohort study, there was no randomization or blinding in our trial, which introduces the potential for selection bias. The decision to proceed with day surgery versus an inpatient admission was made on an individual basis by the principal surgeon. As only one principal surgeon was involved in the decision for day surgery versus inpatient admission, the external validity of the study results may be affected. Patients with medical comorbidities that contraindicated day surgery were allocated to the inpatient group, which may bias the results. Goltz et al. [20] created a predictive patient selection tool for prolonged hospital admission post outpatient shoulder arthroplasty which included factors such as age, sex, cardiac arrhythmia, electrolyte disorder, marital status, ASA, diabetes, and coagulation deficiency—these factors were similar to those used by the senior surgeon when deciding which patients were suitable to be inpatients; however, there were no specific criteria that the senior surgeon used. Pre-operative narcotic usage and mental health were not consciously considered in the decision-making, which may significantly influence the patients’ pain experience post-operatively based on existing literature [19]. It should be noted, however, that the two groups were found to be equally matched with regard to many pre-operative parameters; there were no pre-operative differences between the groups with regard to age, gender, pre-operative pain, and pre-operative function.

The retrospective design also meant patients’ data were collected at different time points for the longer-term follow-up. Data for the first six months after surgery were collected prospectively; however, all the ‘minimum two-year’ follow-up phone calls were conducted in 2013. The time between surgery and this follow-up, therefore, ranged from two years to nine years. The sample size was therefore decreased roughly by half at the two-year follow-up.

One of the strengths of our study was the large sample size of 73 patients, which was one of the largest published studies on the outcomes of outpatient shoulder arthroplasty. While the study was not randomized, statistical analysis reveals that the two cohorts had similar characteristics, with no significant differences in any major demographic categories noted.

## 5. Conclusions

Compared to inpatients, outpatients were not more likely to experience complications, be readmitted to hospital, or require revision surgery. Compared to outpatients, patients who underwent inpatient shoulder arthroplasty experienced significantly less pain three to six months after surgery, and these patients were less likely to seek support from their family physician. Functionally, outpatients performed significantly better than inpatients at several time points and, by six months, had a slightly superior range of motion and strength outcomes than the inpatient cohort. The majority of patients in both groups reported that they preferred future arthroplasty to be done in an inpatient setting. Our outcome findings suggest that although there are minor advantages and disadvantages of performing shoulder arthroplasty as a day case, patients prefer inpatient shoulder arthroplasty.

## Figures and Tables

**Table 1:** Patient demographics (continuous data given as mean ± standard deviation).

	Inpatients	Outpatients	*p*-Value
**Total (n = 73)**	36 (49.3%)	37 (50.7%)	-
**Gender (Male:Female)**	17:19	18:19	1.00
**Age (years)**	69.8 (±10.7)	67.7 (±9.1)	0.53
**Affected shoulder (Right:Left)**	19:17	23:14	0.82
**Procedure (HAS:TSA)**	10:26	12:25	0.65
**Operative time (min)**	93 (±23)	92 (±29)	0.83
**Duration of symptoms (months)**	60.8 (±73.2)	67.3 (±95.5)	0.78

**Table 2:** Post-operative complications in the inpatient group.

Intraoperative Complications	Immediate Post-Operative Complications (<1 Week Postop)	Later Post-Operative Complications (>1 Week Postop)
Intraoperative bleed	Fever	Ruptured long head of biceps
Dysrhythmia	Fever	Shoulder dislocation
	Nausea	
	Nausea	
	Dyspnoea	
	Disorientation	
	Oxygen requirement	

**Table 3:** Post-operative complications in the outpatient group.

Intraoperative Complications	Immediate Post-Operative Complications (<1 Week Postop)	Later Post-Operative Complications (>1 Week Postop)
Surgical bleed	Post-operative pain and bruising	Superficial skin infection
Ulnar nerve palsy	Trapezius discomfort	Joint infection
Radial nerve palsy, Dupuytren’s contracture	Post-operative stiffness	Fell on shoulder two months post-operatively
Dysfunction of wrist extension, elbow flexion, C6/C7 numbness	Hypotension	Recurrent syncope post-operatively
Haematoma		

## Data Availability

The data will be made available in a publicly accessible repository.

## Supplementary Materials

The following supporting information can be downloaded at: https://www.mdpi.com/article/10.3390/jcm12123886/s1.
