# Supplementary material for: Shoulder Arthroplasty as a Day Case: Is It Better?
Source: J Clin Med. 2023 Jun 7;12(12):3886. doi: 10.3390/jcm12123886 (PMC10298933; doi:10.3390/jcm12123886)
Supplement: Supplementary file 1 [file jcm-12-03886-s001.zip › jcm-2413086-supplementary.pdf]

Supplementary Materials- Two year follow up telephone survey questions

- Were you admitted to hospital within 6 months of your operation?
  - If yes, why were you admitted
- After 6 months, were you admitted to hospital for any problems with your shoulder?
  - If yes, why were you admitted
- Have you required any further operations on your shoulder?
- Have you visited your GP for any problems with your shoulder since the operation?
- After 6 months, did you experience any of the following in your shoulder:
  - Severe pain
  - Redness
  - Swelling
  - Numbness or tingling down the arm
- If you had to have the operation again, would you prefer to have it performed as an inpatient or an outpatient procedure?
